# P04.78. Development of an integrative service model for dysthymia patients with body-mind-spirit approach in Chinese medicine clinics in Hong Kong

**DOI:** 10.1186/1472-6882-12-S1-P348

**Published:** 2012-06-12

**Authors:** M Li, C Chan, C Chan, H Chan, L Hui, E Ziea

**Affiliations:** 1 Chinese Medicine Department, Hospital Authority, Hong Kong, Hong Kong; 2 The Centre on Behavioral Health, the University of Hong Kong, Hong Kong, Hong Kong

## Purpose

The prevalence of anxiety and mood disorders in Hong Kong was found to be 4.1% and 8.4% respectively. This study aimed at exploring a sustainable and practical model for incorporation of patient empowerment element, through an integrative Body-Mind-Spirit (I-BMS) approach, for treatment of dysthymia patients in Chinese Medicine (CM) out-patient clinics in Hong Kong.

## Methods

In the first pilot, in addition to routine CM treatment (herbal and acupuncture), CM Practitioners also provided general psychological counseling, advice on dietary regime and self-administered acupressure based on syndrome differentiation for dysthymia. The Centre on Behavioral Health at the University of Hong Kong was commissioned in the second phase to develop a tailor-made I-BMS intervention program with CM concepts. The Centre provided six 3-hours sessions of I-BMS intervention for dysthymia patients recruited. Evaluation included validated questionnaires like the Hospital Anxiety and Depression Scale (HADS) and the Brief Symptom Inventory 18 (BSI-18) for pre-post comparison of clinical outcomes.

## Results

Sixty-six patients participated in the initial pilot and the major CM diagnosis was Bu Mi (insomnia). Fifty-eight patients attended the group intervention sessions in phase II with average attendance rate of 91.8%. Among those who completed the HADS and BSI-18 questionnaires (n=45), there was a significant drop (p<0.01) in domains of anxiety and depression in HADS and BSI scores, which indicated clinical improvement.

## Conclusion

Given resource and manpower considerations in CM clinics, the patient empowerment model in phase II was clinically practical and effective, fostering a synergic effect with CM treatment. The way forward is to integrate I-BMS patient empowerment element into CM service for dysthymia treatment in a “train the trainer” approach. Content of the I-BMS intervention will be consolidated to produce a trainer’s manual for CMPs and a set of patient empowerment material. The resulting service model will be led by CMPs equipped with I-BMS knowledge and skills.

